# Neck Contracture Release With Matriderm Collagen/Elastin Dermal Matrix

**Published:** 2011-03-22

**Authors:** John E. Greenwood, Ian P. Mackie

**Affiliations:** ^a^Adult Burn Centre, Royal Adelaide Hospital, South Australia; ^b^Frenchay Hospital, Bristol, United Kingdom.

## Abstract

**Aims:** To demonstrate success with immediate split-skin graft application over Matriderm dermal matrix in a difficult neck contracture release. **Methods:** An aggressive neck contracture release, accompanied by complete platysmectomy, was followed by application of Matriderm, split-skin graft, Mepitel, and vacuum-assisted closure (VAC) dressing. **Results:** At VAC removal (day 7), graft take was almost complete over the dermal matrix and with minor “touch-up” were complete by day 9 postrepair. Results at 4 months show graft contraction and a marked diminution of the release obtained. The results, however, are still good and the patient is very happy. **Conclusion:** Immediate grafting over a dermal matrix appears to provide a good solution, with a gentle surgical learning curve, in this difficult postburn scenario. Postrelease contraction is, however, as inevitable as with other techniques.

Neck contracture postburn is one of the most difficult burn sequelae to manage. Poor long-term results can follow even extensive release. Disappointing results with split-skin grafting seem to have their roots in 3 phenomena—the natural contractile tendency of grafts, the pain of release that reduces exercise and splintage compliance while the grafts are in their most contractile phase, and the presence of a vestigial remnant of the panniculus carnosus, the platysma.

The effects of this latter structure are tacitly acknowledged but have not been appropriately explained.^1^ Surgeons understand that the panniculus carnosus is a thin muscular layer deep to the skin, within the adventitial tissue of hair-bearing mammals. It is vestigial in humans, remaining as the platysma (with its contributions to risorius and other facial muscles), the dartos muscle of the scrotum, the corrugator cutis ani, the subareolar muscle around the nipple, and the (sometime) palmaris brevis muscle in the hand. In humans, the majority of the panniculus carnosus has been lost with evolution (along with most of our hair) leaving a poor substitute, the fatty panniculus adiposus. In this respect, we resemble the cetacea and domestic pig.^2^

It is likely that it has an important function in nature, which is illustrated by the pathological events during human postburn neck contraction. In a study in which 5 × 5 cm wounds were surgically created in sheep and left to heal spontaneously, full healing took 29 days and left a final healed scar size of 1 × 1 cm (representing a 96% reduction in wound area).^3^ Marked contraction of the panniculus carnosus occurred within a few days of loss of the overlying skin. This contraction reduces wound area and may even appose the wound edges depending on the initial size of the wound. This markedly expedites the rate of healing. Without this facility in the wild, an injured animal, whether predator or prey, would be seriously disadvantaged relative to its counterparts. The panniculus carnosus may represent a chance for survival in such circumstances. This might explain why the panniculus carnosus persists in humans mainly as a thin sheet of muscle covering the tender structures of the neck (platysma), which is the most frequently attacked site by large mammalian predators. Evolutionarily, this might have preserved life after failed attacks with rapid closure of skin over vulnerable and vital structures.^2^

However, rapid closure of any significantly sized wound on the neck brings with it compromise in cosmesis. The origins of the platysma, from the manubrium to acromion inferiorly and the lower border of the mandible superiorly, demonstrate the area over which this typically neglected muscle functions. Contraction, secondary to injury, unfortunately distorts the usual contours of the neck, dulling the inferior mandibular border, obscuring normal surface anatomy, and blunting the cervicomental angle. Complete excision of the platysma to restore neck cosmesis is not a new concept and has been advocated previously.^1^

Obviously, complete excision of platysma necessitates reconstruction of the neck. Typically, this is achieved by split skin grafting, although poor long-term results with reoccurrence of the contracture appear inevitable.

The frustration of surgeons with poor results following skin grafting has led to a stream of alternative maneuvers, which have included large local flaps and free flaps (with or without grafting of the secondary defect), local or distant flap raising after tissue expansion. Some of these techniques have yielded beautiful results in the literature. However, in the developed world, most neck contraction is seen in the setting of very large burn injuries where such flap options are usually limited or nonexistent.

A great deal of excitement followed the introduction of Integra dermal matrix. A material that, when used appropriately, reduces wound contraction by “transforming” a split-skin graft into a full-thickness graft by “creating” a neo-dermis. However, in neck contraction, even this strategy is difficult to employ. In the “cold” setting of burn reconstruction, Integra can take 4 to 5 weeks to vascularize during which it is vulnerable to infection and fragile to mobilization until overgrafted. Thus, some of the extent of release is lost before the grafts are applied.

A newer material, Matriderm (De Suwelack Skin and Health Care AG, Billerbeck, Germany), has been marketed recently.^4^ It is a 3-dimensional dermal matrix composed of bovine dermal collagen (I, III, and V) and bovine nuchal ligament elastin. The manufacturers claim that it can be applied into a debrided wound and split-skin graft applied immediately and that the matrix has hemostatic properties, which reduce the incidence of subgraft and submatrix hematoma. The claim of the ability for immediate application under split-skin graft has been supported by 3 small studies, although study designs precluded comparison with industry standards, like Integra.^5^,^6^ A 1-stage dermal replacement might be able to solve some of Integra's problems.

Our patient is a male, involved in a car rollover and fire, in which he was trapped for some time. He sustained full-thickness burns to face and neck, upper anterior and posterior torso, and both upper limbs completely (35% total body surface area). He had an extremely significant inhalation injury, which necessitated 6 weeks of intensive care unit (ICU) admission, with intubation followed by tracheostomy at 2 weeks. His burns were excised from his upper limbs, chest, back and shoulders immediately on arrival (within 12 hours of his burn injury) and grafted successfully. As is our policy, the facial and neck burns were initially treated conservatively although the head burns were excised 2 days later and grafted. He developed vancomycin-resistant enterococcus (VRE) and methicillin-resistant *Staphylococcus aureus* (MRSA) on ICU and the development of his lower airway chemical pneumonitis precluded further acute intervention. He struggled physiologically for several weeks before being fit for discharge from the ICU to the burns unit.

He already had a degree of neck contracture by that time, but other issues were more pressing and needed intervention, such as bilateral upper and lower eyelid ectropion. His physical state precluded significant major intervention and the residual wound contamination with MRSA and VRE discouraged surgical heroism. He was transferred to our rehabilitation facility for aggressive therapy. It became obvious after a few weeks that his neck contracture was worsening. Options for release and repair were assessed. The degree of contracture (6 cm from point of chin to sternal notch) meant that the created defect after release was going to be very large and a full platysmectomy would be essential (Fig 1). He had no free flaps capable of closing such a defect and no local tissue suitable for expansion. The tension in the neck had caused breakdown of chest and cheek scars, which were MRSA/VRE contaminated, meaning that a 2-stage Integra procedure would be foolish. However, a release was needed that would be more sustainable than split-skin grafting alone.

Matriderm has recently been released onto the global scene although it is not yet registered with the Therapeutic Goods Administration for use in Australia. The offer of a 1-stage dermal matrix and grafting option was appealing, since earlier healing would avoid infective problems and the use of vacuum-assisted closure (VAC) device, as a dressing would allow conformation of material and graft. Following purchase and requisite approval, the operation was scheduled.

## METHODS

**Figure 2:**
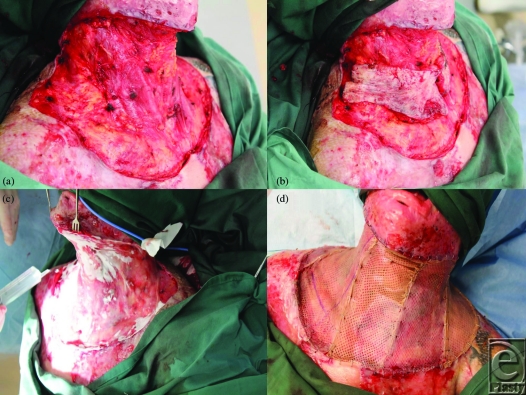
(a) The full extent of the release following skin excision and total platysmectomy. (b) The excised skin is returned to the wound to illustrate the degree of contracture. (c) Matriderm is applied dry into the defect and wetted with saline in situ. (d) The Matriderm is overgrafted with meshed autograft.

**Figure 3:**
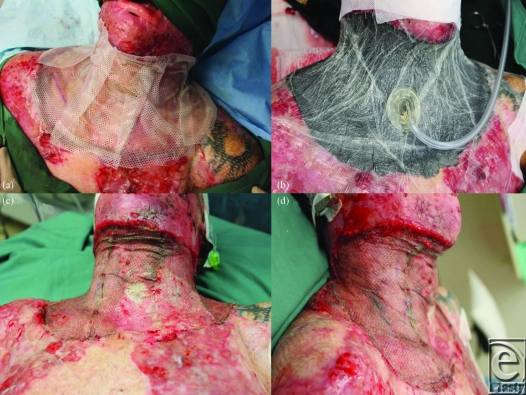
(a) The graft is overdressed with Mepitel. (b) A topical negative pressure dressing holds the arrangement in place. (c) and (d) At 7 days the VAC is removed and the graft inspected.

The neck was marked and then tumesced with our standard solution (1 in 500000 adrenaline and 0.02% bupivacaine). The entire neck skin was excised down to the level of the platysma, from the lower border of the mandible to a subclavicular line and hemostasis secured. The platysma was then raised in its entirety and excised (Figs 2a and 2b). Again, meticulous hemostasis with bipolar diathermy was observed. A single Matriderm sheet (A4 size, 210 × 297 mm) was just large enough to fill the defect. It was applied dry and then wetted with normal saline (Fig 2c). Good-quality split-skin graft had been harvested at 12/1000th of an inch thick from the right thigh and was meshed 1:1 with a noncrushing Brennan mesher before being applied with staples (Fig 2d). The whole was overdressed with Mepitel (Fig 3a) and a standard VAC dressing applied over it and sealed with Opsite (Fig 3b). The pressure was set at −100 mm Hg continuously. Compliance issues forced us to keep the patient intubated on the ICU for 48 hours before he returned to the ward. The VAC dressing was left in place for 7 days.

**Figure 4:**
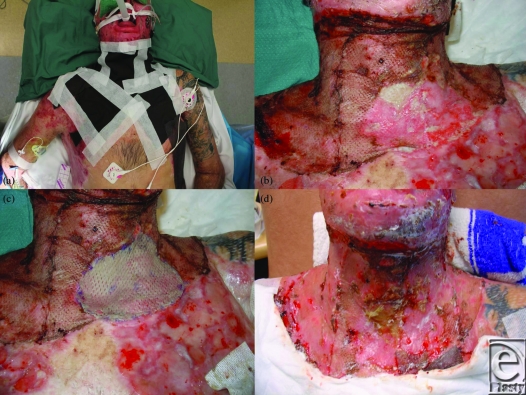
(a) Redressing with Acticoat Flex. (b) At day 9 postrelease, a small area of graft is noted to have failed. (c) Stored skin is used to regraft the defect. (d) By day 12, the new graft has “merged” in and the old graft is quite robust.

At day 7, the VAC was removed. The graft was almost completely taken except for a small area adjacent to a contaminated chest wound (Figs 3c and 3d). After cleaning, Acticoat Flex was applied for 2 days (Fig 4a). At day 9, a small piece of stored skin was applied over the area of graft loss (the Matriderm remained intact at this site) and glued into place with Histoacryl tissue adhesive (Figs 4b and 4c). By day 12, 100% graft take was recorded (Fig 4d). The patient returned to the rehabilitation center for further aggressive therapy.

## RESULTS

**Figure 5:**
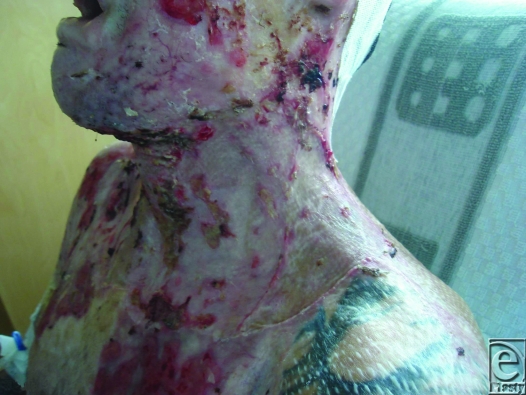
Day 21 postrelease.

**Figure 6:**
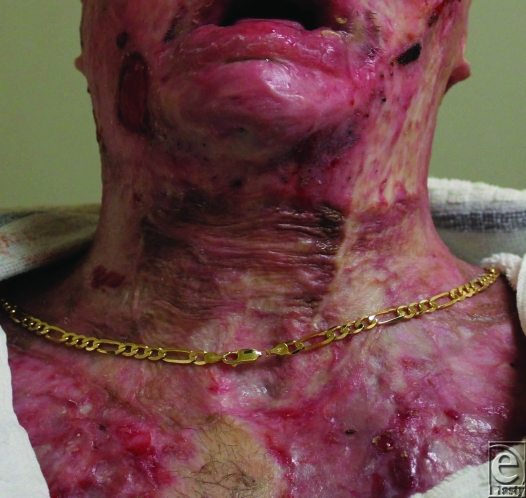
Six weeks postrelease.

**Figure 7:**
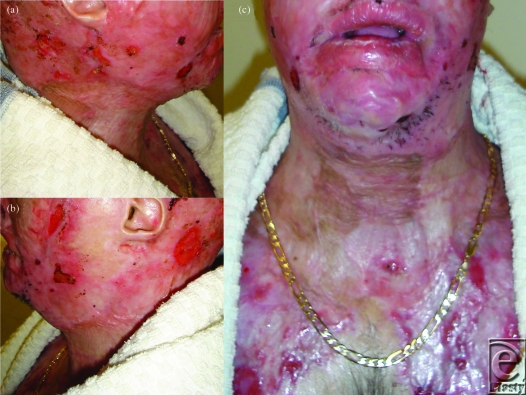
Three months postrelease; result assisted by compression garments.

In a patient with a difficult and severe neck contracture, a full neck release was performed with a 1-stage dermal matrix/skin graft repair assisted by VAC. Full graft take was achieved, with only minimal touch-up over a small graft loss (Figs 5 and 6). The grafted repair rapidly became robust and provided a good early cosmetic result. The early functional result (2 weeks) was far better than preoperatively with 30° lateral flexion, 40° forward flexion and 30° left and right rotation. By 3 months, the cosmetic appearance was excellent, movement was further improved, and the patient was delighted (Figs 7a to 7c).

**Figure 8:**
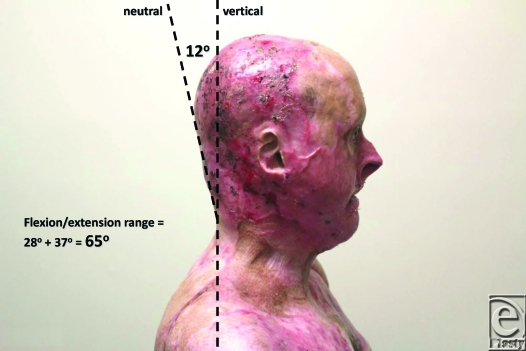
Lateral view, head in neutral. The “plane” of measurement is 12° off the vertical.

**Figure 9:**
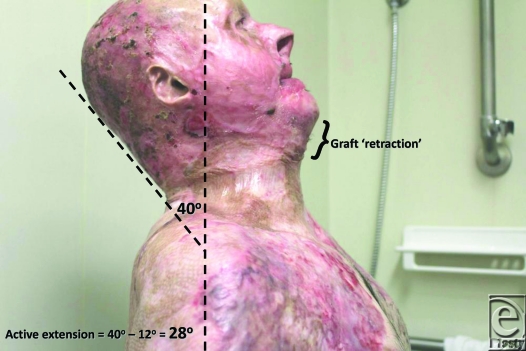
Full extension allows 40° from the vertical (minus 12° neutral) equating to 28° of active extension.

**Figure 10:**
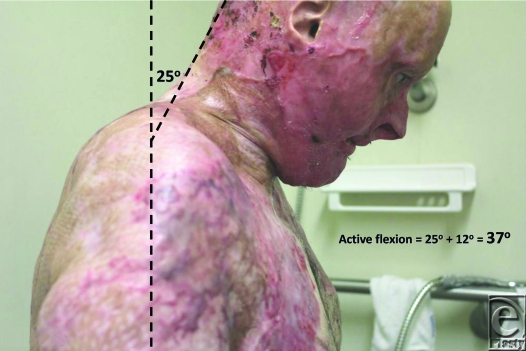
Full flexion allows 25° from the vertical (plus 12° neutral) equating to 37° of active flexion. Total active flexion/extension is thus 65°.

**Figure 11:**
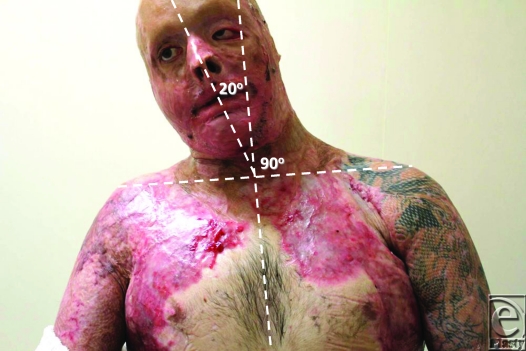
Active right lateral flexion of 20° from neutral.

**Figure 12:**
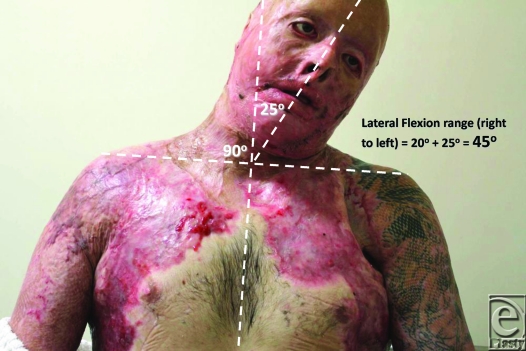
Active left lateral flexion of 25° from neutral. Total active lateral flexion is thus 45°.

**Figure 13:**
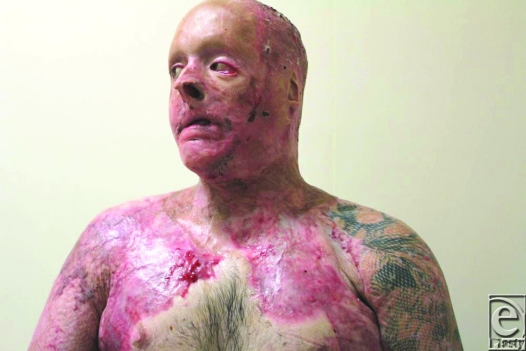
Active right rotation.

**Figure 14:**
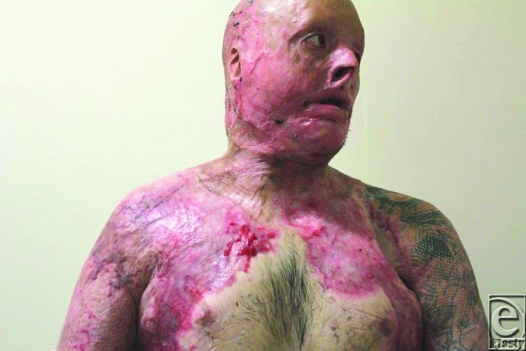
Active left rotation.

**Figure 15:**
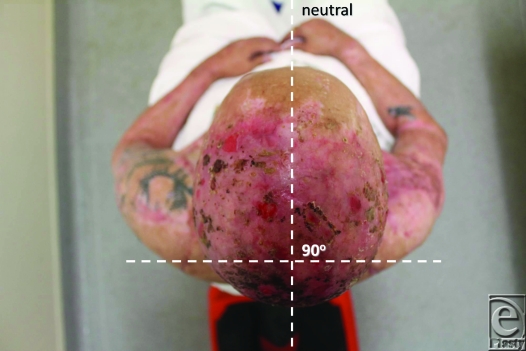
Neutral position for rotation.

**Figure 16:**
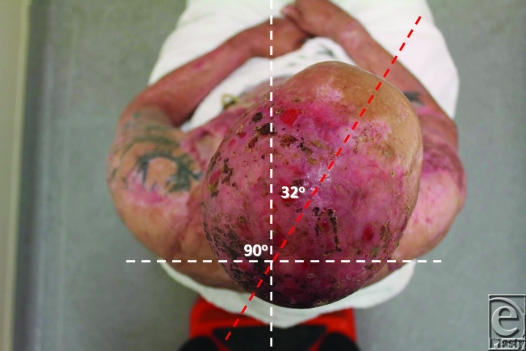
32° of active right rotation.

**Figure 17:**
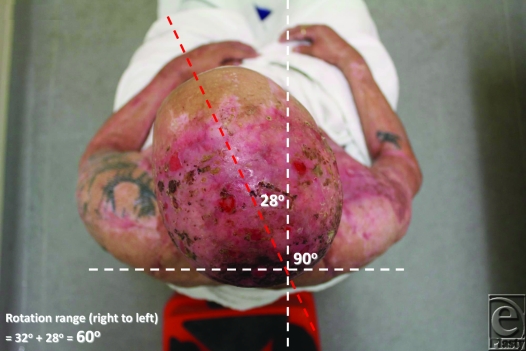
28° of active left rotation. Total active rotation is thus 60°.

Review of outcome was recently performed at 4 months postrelease and repair. At this stage, “normal” scars are generally considered to be the worse they will be, before continuing maturation forces them to settle, flatten, and lose their vascularity. The ranges of movement possible at this 4-month time point are illustrated in Figures 8 to 17. The figures represent active movement and, with passive encouragement, greater ranges were possible. There had been some diminution of ROM in most directions but the patient remained happy. With major facial reconstruction planned in the near future, he believed that the neck restitution provided a good platform for continued reconstruction. We believe that further diminution is unlikely given the “relative” maturity of the grafts. Figure 9 does reveal the degree of early contraction since the original release, and grafting, went from point of chin to sternal notch; graft retraction of several centimeters can be seen in the upper neck (bracketed).

## DISCUSSION

This new material has some significant advantages over Integra, specifically its ability to be applied and immediately overgrafted. The vulnerability of Integra to infection precluded the use of a 2-stage repair. Recent reports document the use of Integra and simultaneous split skin grafting in a single-stage procedure. To date, such practices have been confined to the excision of facial skin cancer in an effort to improve cosmetic and functional result, but involving much smaller wound areas that that created in this case.^7^,^8^ Single-stage Integra and split skin graft has been reported for reconstruction of a radial forearm free flap donor site, again a much smaller defect than that created by the release of significant neck contracture.^9^ That report recommended that both the Integra and skin graft be meshed 1.5:1 to improve the likelihood of graft survival. The resultant mesh pattern may be considered by many to be suboptimal.

The benefit of using Matriderm is, however, marred by the fact that it is as costly as Integra (another case of development, manufacturing, and regulatory costs of a biological material being passed on to the end user). From a technical perspective, our first use of this material, possibly in the most difficult indication imaginable, has been highly successful, indicating that the learning curve for this material is very gentle (certainly compared to Integra). Four-month results indicate that this repair is not without subsequent contraction and, although the result illustrated by Figures 8 to 17 is far better than his prerelease state, it is disappointingly different from the immediate postrelease result. Figure 9 illustrates in the lateral view how far the repair “retracted” from its immediate submental position. I have no doubt that this result is better than our previous Integra releases because of the ability to start therapy at an earlier stage, but such contraction is disappointing given the magnitude of the release. We await the long-term result (12 – 24 months) with interest and expectation.

## CONCLUSION

Matriderm is a relatively new introduction into the burn surgeon's arsenal. It appears easy to use with a little forethought, even in difficult situations. Obviously, wetting it before application leaves one wrestling with a fragile sheet akin to wet tissue paper and placement before wetting is advisable. Although some long-term data are published, success in specific situations such as neck contracture release is lacking. Contraction does occur, although the facility for earlier therapy seems to make this less than 2-stage reconstructions.

## Figures and Tables

**Figure 1:**
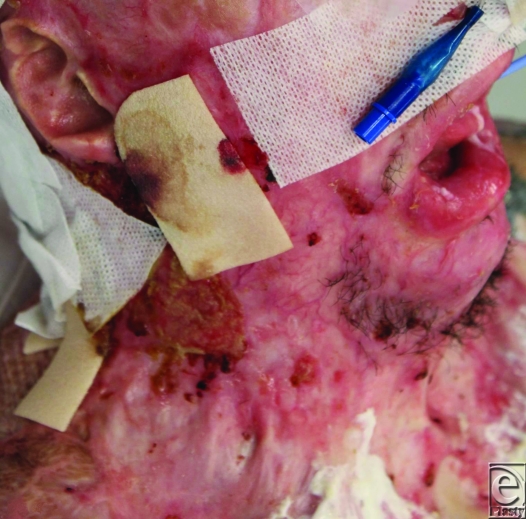
Preoperative view of the neck contracture.
